# Taking stock of cost-effectiveness analysis of healthcare in China

**DOI:** 10.1136/bmjgh-2019-001418

**Published:** 2019-05-14

**Authors:** Thomas Butt, Gordon G Liu, David D Kim, Peter J Neumann

**Affiliations:** 1 National School of Development, Peking University, Beijing, China; 2 Institute of Ophthalmology, University College London, London, UK; 3 Center for the Evaluation of Value and Risk in Health, Tufts Medical Center, Boston, Massachusetts, USA

**Keywords:** cost-effectiveness analysis, health technology assessment, QALY, DALY, China

## Abstract

**Introduction:**

Cost-effectiveness analysis (CEA) is playing an increasingly important role in informing healthcare decision-making in China. This study aims to review the published literature on CEA in mainland China and describe its characteristics and evolution. We provide recommendations on the future direction of CEA as a methodology and as a tool to support healthcare decision-making in China.

**Methods:**

English-language cost-per-quality-adjusted life-year (QALY) and cost-per-disability-adjusted life-year (DALY) publications relating to mainland China were reviewed using the Tufts Medical Center Cost-Effectiveness Analysis Registry and Global Health Cost-Effectiveness Analysis Registry through 2017. Study features were summarised using descriptive statistics. Changes in study methodology over time were analysed by trend test, and study characteristics influencing the incremental cost-effectiveness ratio (ICER) of cost-per-QALY studies were investigated using logistic regression.

**Results:**

170 studies were identified reporting CEA for mainland China (cost/QALY=125, cost/DALY=45) since 1998. The number and quality of studies has increased over the past two decades, with significantly more cost-per-QALY studies compared with cost-per-DALY studies (p<0.0001) and more studies with authors affiliated with Chinese institutions (p=0.0002). The average quality score was 5.04 out of 7 for cost-per-QALY and 4.70 for cost-per-DALY studies based on Registry reviewers’ subjective assessment of overall quality (methods, assumptions and reporting practices). The median ICER reported for interventions for oncology patients was higher (US$26 694 per QALY) than the median ICER reported for all interventions (US$11 503 per QALY). Oncology interventions were associated with the likelihood of reporting higher ICERs than the median ICER (p=0.003).

**Conclusion:**

The number of English-language published CEA studies relating to China has grown rapidly over the past 20 years. In terms of quality, the China studies compare favourably with international studies, although they remain a small proportion of studies globally.

**Key questions**

**What is already known?**

- Prior reviews reported a small body of good-quality cost-effectiveness analysis (CEA) studies relating to China.
- They identified relatively few studies compared with our extractions from the Tufts Medical Center Cost-Effectiveness Analysis Registry and Global Health Cost-Effectiveness Analysis Registry and they were limited in their comparisons with the international CEA literature.

**What are the new findings?**

- This review found an expanding body of well-constructed cost-per-quality-adjusted life-year and cost-per-disability-adjusted life-year studies relating to mainland China.
- There were some unique features of the CEA literature for China compared with the international CEA literature such as a different sponsorship pattern, with the government funding a higher proportion of studies.

**What do the new findings imply?**

- The profile of CEA is growing in China although the number of published studies remains a small proportion of studies published globally.

## Introduction

Cost-effectiveness analysis (CEA) quantifies the incremental costs and benefits of interventions compared with alternative uses of resources, supporting judgements on whether health technologies provide good value.^1^ Faced with competing demands for limited healthcare budgets, CEA is increasingly used to inform healthcare decision-making around the world. Examples of national health technology assessment (HTA) agencies include the National Institute for Health and Care Excellence in the UK, the Canadian Agency for Drugs and Technologies in Health in Canada and the Agency for Care Effectiveness in Singapore.^2–4^

Several studies have reviewed the international CEA literature, finding a rapid growth in studies and the expansion of the methodology to non-Western countries.^5 6^ In China, there is a desire to move towards a data-driven healthcare system with China’s national health strategy^7^ ‘Healthy China 2030’ setting specific indicators to measure success^7^; in addition, a number of health policy reforms have promoted HTA and comparative effectiveness research in recent years.^8^ Guidelines for conducting pharmacoeconomic evaluations in China were first published in 2011 and are updated as methods develop.^9^

However, previous reviews have identified relatively small numbers of published CEA studies relating to China. A systematic review of pharmacoeconomic studies in the Asia-Pacific region included 128 studies, but none of the included studies were conducted in China.^10^ Another systematic review of pharmacoeconomic publications produced in China between 2006 and 2012 included 20 studies.^11^ While another systematic review of the quality of pharmacoeconomic publications from China through 2014 included 32 studies.^12^

Two special cases of CEA, which differ in their quantification of health benefit, have become increasingly prominent in recent decades. The first uses the quality-adjusted life-year (QALY) as a measure of health benefit. The QALY weights each year of life lived using a preference for health state (utility) anchored so that 1 represents perfect health and 0 is equivalent to the state of being dead.^13^ Many national HTA agencies recommend the number of QALYs gained as a unit of benefit for evaluations.^3^

The second case uses the disability-adjusted life-year (DALY) as a measure of health benefit. The DALY weights years of life using equivalent years of ‘healthy’ life lost by virtue of being in states of poor health or disability. The DALY is frequently used when assessing the impact of public health interventions with benefit in this case expressed in terms of DALYs averted. The WHO uses the metric in its Global Burden of Disease Study.^14^

This study aims to review the published literature on CEA (cost per QALY gained and cost per DALY averted) of health technologies in mainland China to describe its characteristics and evolution in order to make recommendations on future directions for researchers and policymakers.

## Methods

### Cost-Effectiveness Analysis Registry

The Tufts Medical Center Cost-Effectiveness Analysis Registry (CEA Registry) is a curated database with detailed information on over 7200 cost-per-QALY gained studies and 18 000 incremental cost-effectiveness ratios (ICER) published in the English-language peer-reviewed literature since 1976. While search strategy and methodology for the Registry have been described previously^6^ and made available in the online supplementary appendix, here we provide a summary. The CEA Registry only contains information from the original studies reporting ICERs (ie, excluding review, editorial or methodological articles). Followed by literature search, at least two reviewers independently conduct a series of screening. After a consensus meeting to determine whether each study is suitable for full or partial or no data collection, data are extracted from each accepted study and made available on the CEA Registry, including the following key variables: country, disease, intervention description, intervention type and comparator description.

10.1136/bmjgh-2019-001418.supp1 Supplementary data

The Global Health CEA Cost-Effectiveness Analysis Registry (GH CEA Registry; www.ghcearegistry.org) follows a similar methodology to the CEA Registry to identify cost-per-DALY studies. The GH CEA Registry has over 600 articles containing cost-per-DALY averted estimates and over 5000 ratios published in the peer-reviewed literature since 1995. For this study, articles that included mainland China (excluding Hong Kong, Macau and Taiwan) as a primary or secondary geographic region were extracted from both the CEA Registry and GH CEA Registry on 17 May 2018.

### Patient and public involvement

Patients were not involved in this research.

### Variables

Data are collected for each article on 40 variables, including author affiliation, study country, study sponsorship, a description of each ratio’s intervention, comparator, target population, the methodology used to measure costs and health utilities, and methods used to calculate cost-utility ratios. All sources of sponsorship and all author affiliations are recorded, meaning that a single study may have several sponsorships or author affiliations. The quality score available from the CEA/GH CEA Registry is assigned by two highly trained Registry reviewers after a consensus meeting. The score is assigned on an interval scale from 1 (low) to 7 (high) based on the following criteria:

- Methods and results were communicated clearly and transparently to enable easy interpretation.
- Time horizon was of sufficient length and discount rate appropriate.
- Detailed disaggregated cost and QALY information was provided and recalculated ICER was correct (or did not differ by more than 10%).
- Comprehensive characterisation of uncertainty (sensitivity analyses).
- Explicit reporting of utility weights (includes utility weight value and estimation method).
- Subgroup analysis performed.
- Non-health effects and/or spillover effects were quantified.

In addition to the standard variables contained within the registry, for included studies we extracted two additional variables: (1) the country of first author affiliation and (2) the cost-effectiveness threshold used to determine if the intervention was likely to be cost-effective.

### Analysis

Descriptive statistics were used to summarise key features of cost-per-QALY and cost-per-DALY studies in China. We further categorised studies featuring China, as having either a primary or secondary geographic focus. The geographic focus refers to the country to which the study’s results are applied. For example, if the primary country is defined as China, then the ICER represents the expected costs and QALYs if interventions were adopted in China. If the primary country is defined as another country, but the study includes a subanalysis for China, then the study is defined as having a secondary geographic focus of China. This differentiation is particularly relevant for cost-per-DALY studies that often cover multiple low and middle-income countries in a single evaluation. Studies were grouped by year of publication to describe study characteristics at different time points.

The Cochran-Armitage trend test was used to investigate trends over time. The association between study characteristics (study sponsors and first author affiliation and the time of publication) was tested.

ICER analysis focused on cost-per-QALY studies with a primary geographic region of China. Cost-per-DALY studies were excluded because the different benefit measures cause non-comparability issues, and the sample of cost-per-DALY studies with a primary geography of China was relatively small for meaningful investigation of their characteristics.

We conducted a statistical analysis to examine the association between study characteristics and the probability of being reported ICER is greater (or lower) than the overall median ICER. Analysis was limited to those ICERs that fell in the north-east quadrant (intervention more costly and more effective than comparator). Using a logistic regression, the dependent variable was a dichotomous variable on whether the reported ICER (in 2017 US$) was higher than the median ICER. Independent variables included disease area (oncology vs not oncology), study sponsor (industry vs non-industry), author affiliation (industry vs non- industry) and study quality (1–7 interval scale).

Each study may report one or more ICERs, which were defined by three characteristics: (1) the target population, (2) the intervention, and (3) the comparator. This means no two ratios reported for the same paper should have identical entries for all three of these characteristics. If two values are reported for the same ratio (ie, the same three elements), the two ratios differ because of an alternative assumption (eg, the use of a societal perspective rather than a healthcare system perspective). Each ratio was assigned a weight of 1/n, where n was the number of ratios reported in an article to ensure that no single article disproportionately affected the results.

Statistical analysis was performed in Stata V.14 (StataCorp).

## Results

One hundred and seventy CEA studies with a geographic focus on mainland China were identified (QALY=125, DALY=45). Table 1 describes that the main study characteristics and study references may be found in the online supplementary appendix. There was a marked difference in the scope of studies: 121 out of 125 (97%) cost-per-QALY studies had a primary focus on China and the remaining four studies included China as a secondary focus. Only 23 out of 45 (51%) cost-per-DALY studies had a primary focus on China and the remaining 22 studies included China as a secondary focus.

**Table 1:** Study characteristics

Variable	QALY		DALY	
	All	Primary geography=China	All	Primary geography=China
Studies, n	125	121	45	23
Top three disease areas, name (%)				
1	Oncology (34.7)	Oncology (35.7)	Infectious and parasitic diseases (62.2)	Infectious and parasitic diseases (60.9)
2	Infectious and parasitic diseases (25.7)	Infectious and parasitic diseases (25.5)	Endocrine, nutritional and metabolic diseases (11.1)	Endocrine, nutritional and metabolic diseases (13.0)
3	Endocrine, nutritional and metabolic diseases (14.9)	Endocrine, nutritional and metabolic diseases (15.3)	Oncology (6.7)	Oncology (8.7)
First author affiliation=China, %	80.8	82.6	35.6	65.2
Academic author, %	79.2	79.3	82.2	58.3
Government sponsorship, %	42.4	42.1	51.1	41.7
Healthcare payer perspective, %	57.1	58.4	35.6	26.1
Quality score, mean	5.04	5.05	4.70	4.41

Disease area categories are based on International Classification of Diseases 10th Revision (ICD-10) chapters.

DALY, disability-adjusted life-year; QALY, quality-adjusted life-year.

**Figure 1:**
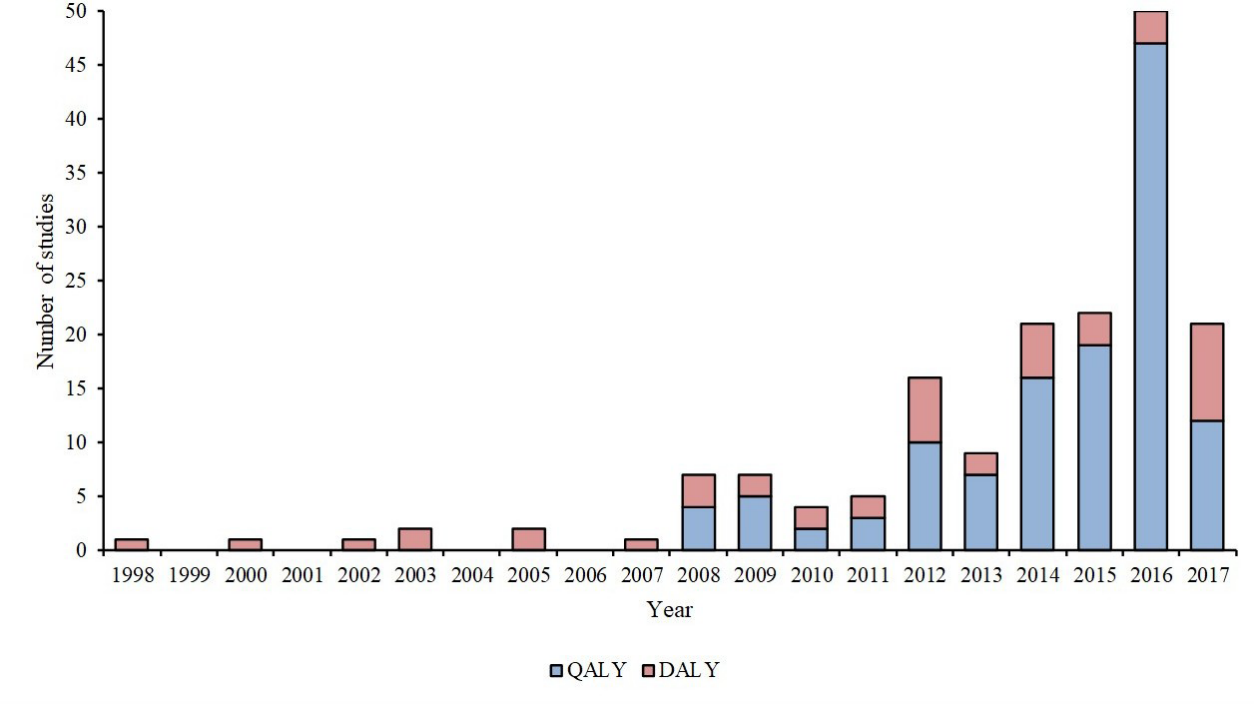
Cost-effectiveness analysis (CEA) studies by year of publication. DALY, disability-adjusted life-year; QALY, quality-adjusted life-year.

Figure 1 shows that the number of studies published has grown over the past 20 years. The first cost-per-DALY study was published in 1998 and the first cost-per-QALY studies 10 years later in 2008. Since 2008, cost-per-QALY studies have dominated the China CEA literature and have accounted for 50% or more of the CEA studies published and reached a high of 94% of CEA studies in 2016. Table 2 shows the proportion of studies with certain characteristics over time. The table shows that the proportion of studies using QALY as the unit of health benefit, the proportion of studies with first author affiliated with an institution in China and the mean subjective quality score all increase over time. The Cochran-Armitage trend test was significant for the proportion of studies using QALYs (p<0.0001) and the proportion of studies with first author affiliated with an institution in China (p=0.0002).

**Table 2:** Changes over time in study characteristics

	1998–2010 (n=26)	2011–2013 (n=31)	2014–2015 (n=43)	2016–2017 (n=71)	Test for trend, P value
QALY as unit of health benefit, %	42.3	64.5	81.4	83.1	<0.0001
First author at Chinese institution, %	38.5	73.3	67.4	78.9	0.0002
Quality score (cost-per-QALY studies), mean	4.59	4.95	4.95	5.27	–
Quality score (cost-per-DALY studies), mean	4.43	4.60	4.81	5.04	–

DALY, disability-adjusted life-year; QALY, quality-adjusted life-year.

### Disease area

Disease areas were classified by the 21 International Classification of Diseases 10th Revision chapters. The top three disease areas covered by cost-per-QALY studies were oncology (35%), infectious and parasitic diseases (26%) and endocrine, metabolic and nutritional diseases (15%). Other disease areas accounted for 25% of cost-per-QALY studies in the Registry, and less than 10% individually.

The top three disease areas covered by cost-per-DALY studies were the same as for cost-per-QALY studies, although in different orders and proportions. They were infectious and parasitic diseases (62%), endocrine, metabolic and nutritional diseases (11%) and oncology (7%). Other disease areas accounted for 20% of cost-per-DALY studies in the Registry, and less than 5% individually.

### Authorship

The location of authors varied between the cost-per-QALY and cost-per-DALY studies. For QALY studies, 101 (81%) of first authors were affiliated with institutions in mainland China. In contrast, 16 (36%) of DALY studies had a first author affiliation in mainland China. The difference may be explained by the different scope of QALY and DALY studies: when only studies with a primary focus on China were included, 100/101 (99%) of QALY studies and 15/16 (94%) of DALY studies were authored in China.

**Figure 2:**
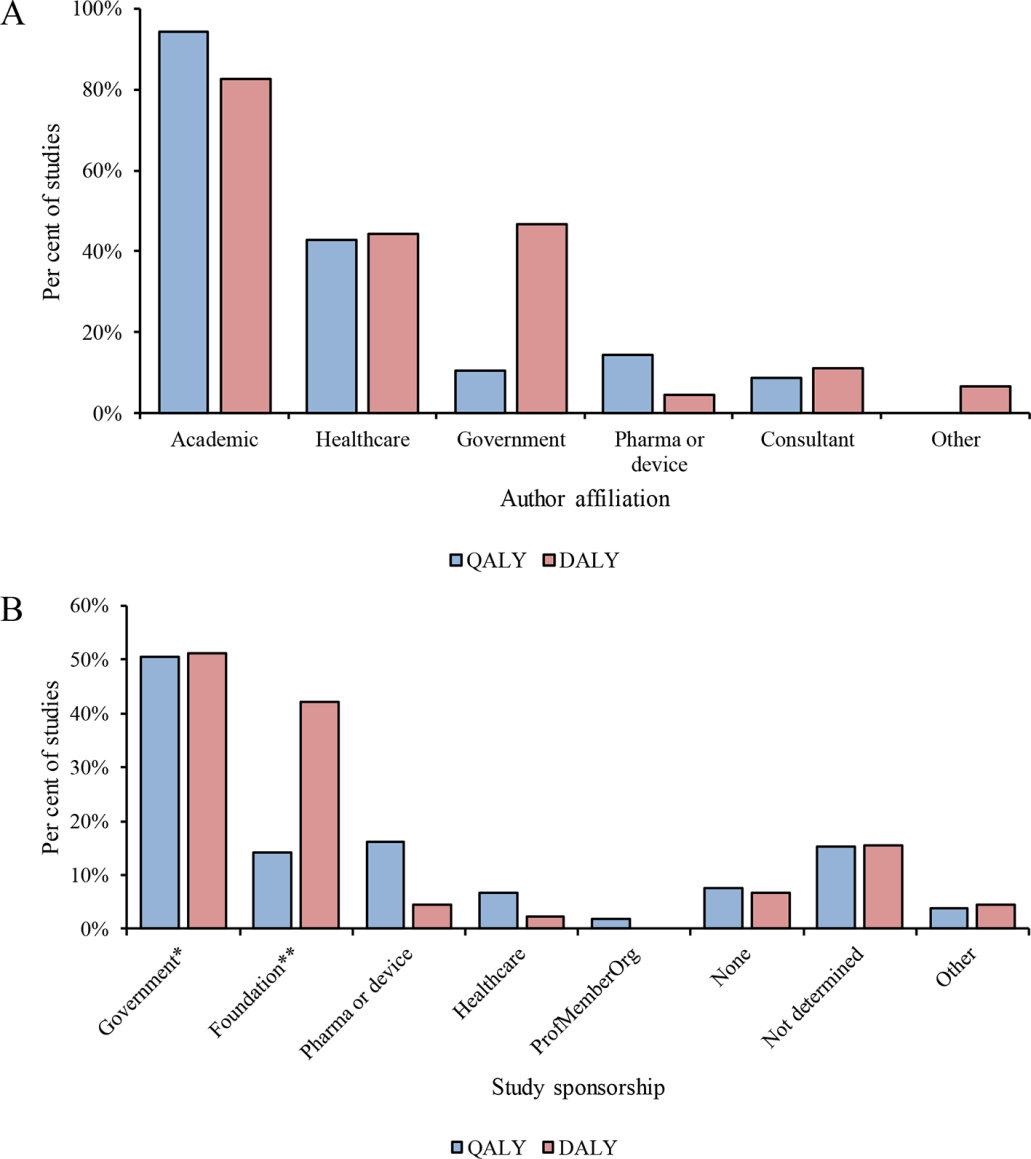
Characteristics of cost-effectiveness analysis (CEA) studies. (A) Author affiliations. (B) Source of sponsorship. *Includes academic. **Includes intergovernmental organisations. DALY, disability-adjusted life-year; QALY, quality-adjusted life-year.

Figure 2 shows the affiliations of all authors of cost-per-QALY and cost-per-DALY studies. Most cost-per-QALY and cost-per-DALY studies included authors affiliated with academic institutions (99, 55%), followed by healthcare institutions (45, 25%). Authors with government, industry and consultancy affiliations each accounted for less than 10% of studies.

### Study sponsorship

As depicted in figure 2, academia and government were sponsors of approximately half of cost-per-QALY and cost-per-DALY studies. Industry sponsored 17 (16%) of the cost-per-QALY studies. Of these studies, no single company was a dominant sponsor, with top sponsors AstraZeneca, Sanofi and Novo Nordisk each accounting for three studies. Only 2 (4%) of cost-per-DALY studies were sponsored by industry, while foundations and intergovernmental organisations sponsored 19 (41%) studies.

### Quality

The mean subjective quality score was 5.04 (SD 1.17) out of 7 for cost-per-QALY studies. This compares favourably with international studies, which averaged a score of 4.20 in a previous report from the same database, although it should be noted that the general quality of CEA studies may have improved between our analysis and this earlier publication, and that the quality score is a relatively crude measure.^5^ The mean subjective quality score was 4.70 (SD 0.97) for cost-per-DALY studies, which was slightly lower than the average of 4.89 reported for a previous study using the GH CEA Registry.^15^ Online supplementary appendix table A1 reports additional key criteria of the quality of cost-per-QALY and cost-per-DALY studies. These criteria were consistent with those that have been reported in previous publications using the CEA Registry to aid comparison. Overall, cost-per-DALY studies scored lower quality than cost-per-QALY studies, but the difference is particularly notable when examining the proportion of studies conducting incremental CEA correctly. While 80.0% of cost-per-QALY studies conducted the incremental CEA correctly, this proportion was only 57.8% for cost-per-DALY studies.

### Incremental cost-effectiveness ratio

There were 296 ICERs for 121 cost-per-QALY studies with a primary geographic region of China. Of these 296 ICERs, 208 (70%) were in the north-east quadrant, meaning that interventions were more effective but more costly than the comparator, with a mean ICER of US$77 138 per QALY (SD: US$243 162) and a median of US$11 503. There were 52 ICERs for oncology interventions in the north-east quadrant and these had a mean ICER of US$118 505 per QALY (SD: US$228 438) and a median of US$26 694. Online supplementary appendix figure A1 shows these ICERs on a cost-effectiveness plane. A further 33 ICERs fell into the northwest quadrant (less effective, more costly/dominated), 8 in the southwest quadrant (less effective, less costly) and 47 in the southeast quadrant (more effective, less costly/dominant).

Table 3 reports the results of logistic regression of the ICERs of cost-per-QALY studies in the north-east quadrant of the cost-effectiveness plane. Model 1 shows that ICERs for oncology, the most common disease area, were likely to be higher than ICERs for other disease areas (OR 3.03, 95% CI 1.47 to 6.27). After including the additional variables for study quality, pharmaceutical industry authorship and pharmaceutical industry sponsorship, model 2 shows that, controlling for quality (subjective quality score), ratios for oncology studies remained likely to be higher (OR 3.22, 95% CI 1.49 to 6.98). Neither authorship nor sponsorship by industry was associated with the chance of an ICER being higher than the median.

**Table 3:** Logistic regression to identify factors that influence the probability that the ICER is greater than the weighted median for ratios in the north-east quadrant for cost-per-QALY studies

	Model 1: disease area			Model 2: full		
**Variable**	**OR**	**SE**	**P value**	**OR**	**SE**	**P value**
Disease area=oncology	3.03***	1.12	0.003	3.22***	1.27	0.003
Sponsor=industry	–	–	–	0.66	0.38	0.466
Author=industry	–	–	–	0.43	0.39	0.352
Study quality (subjective score)	–	–	–	1.27*	0.17	0.069
Constant	0.70*	0.15	0.086	0.21**	0.15	0.026
Observations	208			208		

Variables were coded as follows: disease area oncology=1, disease area not oncology=0; study sponsor included industry=1, sponsor did not include industry=0; author list included industry=1, author list did not include industry=0; study quality=1–7 interval scale (half-points were possible).

***p<0.01; **p<0.05; *p<0.10.

ICER, incremental cost-effectiveness ratio; OR, odds ratio; QALY, quality-adjusted life-year; SE, standard error.

Studies applied a variety of cost-effectiveness thresholds to determine if the intervention was likely to be cost-effective. The most commonly used threshold was three times country-level gross domestic product (GDP) per capita used in 59 (49%) of the 121 cost-per-QALY studies with a primary geographic focus on China. GDP-based thresholds including one time, two times and ranges of 0–3 times GDP per capita were applied in 30 studies (25%) and a further 4 studies (3%) used other thresholds. Eleven studies (9%) used a regional or city GDP per capita as the threshold. Seventeen studies (14%) failed to report a threshold.

## Discussion

This study identified the largest body of CEAs relating to China to date and compared it with the international CEA literature. The number of publications remains low by global standards, with just 170 studies identified. Proportionally, Chinese cost-per-QALY studies represent only 1.7% of the CEA Registry, reflecting the fact that historically the QALY has been the most commonly applied to high income settings.^15^ Chinese cost-per-DALY studies represent a larger share of 7.7% of the GH CEA Registry.

We hypothesise several reasons for the relatively low number of published CEA studies relating to China. First, some articles may be published in Chinese-language journals, which are not recorded in the CEA Registry nor international indexing services such as PubMed. Second, government institutions conducting CEA studies may not choose to publish their analysis due to a political environment where decision-making process remains largely confidential with limited transparency. Grey literature reports and dossiers that may be used in healthcare decision-making but were not published in the peer-reviewed literature and excluded from our study. Finally, a formal HTA agency for China was only established in October 2018. Previously the lack of a HTA agency in China to encourage the provision of economic evidence may have limited public and private funding for CEA research. Neumann *et al* found that the top countries publishing (English-language) cost-effectiveness studies across all types of sponsorship were the USA, UK, Canada, the Netherlands and Sweden. The latter four countries have well-established HTA agencies which recommend cost-per-QALY analysis, and interest in these studies to support decision-making in the USA is growing.^16^

CEAs focused on China are mainly published by authors affiliated with Chinese institutions. This trend is expected to continue as the number and size of health economic research groups at Chinese universities continues to grow and government policies encourage comparative effectiveness research.^17^

The QALY continues to establish itself as the preferred unit of health benefit for CEA in China among published CEAs and there has been rapid growth in cost-per-QALY studies in recent years. The first three cost-per-QALY studies were published in 2008, we do not believe this was associated with any particular local political or methodological development. More recently, the EQ-5D value set for China was published in 2017, which enables QALYs to be estimated that reflect the preferences of the local Chinese population, removing an important limitation of earlier cost-per-QALY studies conducted in China.^18^ As infectious diseases still pose substantial disease burden in China and many of China-based cost-per-DALY studies focused on these areas, cost-per-DALY studies could also provide important information for priority settings among public health interventions in China.

The pharmaceutical industry is a relatively minor sponsor of cost-per-QALY studies in China when compared with international trends (14% in China vs 24% internationally reported by Neumann *et al*^5^). These differences may reflect the lack of explicit guidance or mandate from the Chinese government that companies should provide cost-per-QALY evidence for pricing and reimbursement decision-making.

Our finding that ICERs relating to oncology studies were likely to be higher than the median ICER was similar to other studies and highlights that there may be systematic differences between cost-effectiveness estimates across disease areas.^19^ Given that there is no agreed cost-effectiveness threshold for decision-making in China, these differences may have important implications for resource allocation.

There appeared to be no influence of industry on the level of the reported ICER of cost-per-QALY studies in contrast to a previous publication using the entire CEA Registry, which found that industry-sponsored studies were likely to have ICERs lower than the median.^20^ That said, the sample of industry-authored and industry-sponsored studies is currently small in China, so it would be interesting to replicate the analysis in the future with a larger sample.

Our finding that CEA studies in China are of a generally high quality is in line with previous publications. Jiang *et al* found that China-based English-language studies had an average Quality of Health Economic Studies scale score of 80 out of 100, and we found that cost-per-QALY studies were of higher quality than previous studies using the CEA Registry and cost-per-DALY studies were of comparable quality to previous studies using the GH CEA Registry.^5 11 15^ Of the 20 studies identified by Jiang *et al*, 11 were also in our review and had a mean score of 4.9 in the CEA Registry quality rating. The nine studies not included in our review were due to Chinese language (2) non-QALY/DALY unit of benefit (2) not mainland China (3) and no mention of QALY/DALY in title or abstract (1).

This study has several limitations. The CEA Registry focuses on two particular forms of CEA, often called cost-utility analysis (CUA): cost-per-QALY gained and cost-per-DALY averted studies. Other forms of economic evaluation that employ other benefit measures, such as disease-specific outcome measures or cost-benefit analysis, are not included in this study. The restriction on CUAs allows more robust methodological and quality comparisons across the included studies.

Second, the CEA Registry indexes English-language literature and therefore publications in local language peer-reviewed journals were not included in this review. While the inclusion of Chinese-language publications might be expected to increase the number of articles identified, the use of the CEA Registry provides a rigorous and standardised methodology identifying and classifying CEA literature, facilitating comparisons between international studies.

Furthermore, the number of studies identified in this review compares favourably with previously published systematic reviews of the Chinese CEA literature.^11 12 21^ The most recently published review by Ma *et al*, which focused on study quality, included 32 pharmacoeconomic studies published up to 2014.^12^ Their review covered additional regions (studies relating to Special Administrative Regions, such as Hong Kong, were included) and also covered measures of effectiveness aside from the QALY or DALY. Over the same time period, our review included 78 cost-per-DALY or cost-per-QALY studies for mainland China.

In light of the increasing importance of CEA in China, we make a number of recommendations for policymakers dealing with the limited evidence base. A challenge in the interpretation of CEAs in China is the absence of an agreed-upon national ICER benchmark or threshold. This is in part due to regional inequality, which means that any standard (eg, one-time GDP per capita) would have different implications for different parts of China, where drug budgets are devolved to local provinces.^22^ We found that most studies were using national GDP per capita to determine a cost-effectiveness threshold.

Although GDP per capita-based thresholds were originally recommended by the WHO, they were not empirically derived.^23^ Many researches have argued that thresholds should reflect the opportunity cost of health, which has not been estimated for China to date.^24^ Decisions based on a GDP-per-capita threshold may lead to inappropriate recommendations as well as misallocation of resources,^25^ and further research into appropriate thresholds for China would be valuable.

Evidence gaps have been noted in the CEA literature, with studies covering pharmaceutical interventions for conditions such as oncology and HIV relatively over-represented, whereas health education interventions and conditions such as injuries and substance abuse are relatively under-represented.^26^ The disease areas we identified as the most common focus of CEA studies in China (oncology, infectious and parasitic diseases, and endocrine, nutritional and metabolic diseases) had some similarities with the leading causes of morbidity and mortality in China, suggesting that the cost-effectiveness literature for China is focused on areas relevant to policymakers. The Global Burden of Disease Study identified cerebrovascular disease as the leading cause of years of life lost (YLL) in 2013, whereas diseases of the circulatory system was the topic of only 9% of cost-per-QALY studies and 4% of cost-per-DALY studies in China.^27^ Cancer, the leading topic of cost-per-QALY studies and third most common topic of cost-per-DALY studies, has been a growing cause of YLL in the Global Burden of Disease Study and is the leading cause of death in China’s national statistics.^27 28^ A more detailed analysis of evidence gaps in China may be valuable to guide limited financial and human resources to conduct the most valuable economic evaluations.

While policymaker’s desire to incorporate economic evidence into coverage decision has been growing in China, there is a need to apply economic evidence generated elsewhere to local settings due to the lack of available evidence specific to China. Further efforts to evaluate transferability of findings would be necessary based on potential differences in population characteristics, disease epidemiology, relative prices, health systems and other factors. The development of an explicit reference-case CEA is also highly recommended to help comparisons across studies, as the conclusions of studies are sensitive to the features of the local healthcare system and societal preferences.

A Chinese-language CEA Registry would be helpful for policymakers to keep track of the Chinese CEA literature in a similar way to the international literature. Over the longer term, policies to encourage open publication of CEA studies in the peer-reviewed literature and development of domestic research capacity to undertake studies will help fill the evidence gaps.

## Conclusion

The body of CEA literature relating to China has grown rapidly in recent years, though the number of published studies remains a small proportion of studies published globally. In light of the growing importance of CEA to policymakers, there is a need to critically interpret evidence from different geographies and settings by adapting models or input parameters to the China situation. Policies to encourage the peer-reviewed publication of analysis and to support capacity building in domestic health economic research are recommended.
