# Supplementary material for: Taking stock of cost-effectiveness analysis of healthcare in China
Source: BMJ Glob Health. 2019 May 14;4(3):e001418. doi: 10.1136/bmjgh-2019-001418 (PMC6528776; doi:10.1136/bmjgh-2019-001418)
Supplement: Supplementary data [file bmjgh-2019-001418supp001.pdf]

Butt T, Liu GG, Kim DD, et al. Taking stock of cost-effectiveness analysis of healthcare in China: Supplemental material

Appendix

Detailed methods for extracting and validating data in the CEA Registry

Abstracts from articles identified by the literature search are screened to determine if the paper contains an original cost-utility estimate. We exclude review, editorial, or methodological articles, as well as cost-effectiveness analyses that do not use QALYs to quantify health benefits.

Abstracts undergo a second screening stage wherein the CEA Registry team selects abstracts for full data collection, partial data collection, or rejection. Articles that are selected to continue on to the next phase of full data collection are those with large disease prevalence, focused on novel treatments, have far-reaching public health implications, include an active comparator, and are easily generalizable across settings. Examples of articles that are selected only for partial review (these articles will not move into the next full data collection phase) include: dose adjustment studies, studies with very small or non-replicable patient populations, and telemedicine interventions for rural populations. Articles that meet any exclusion criteria outlined during the initial screening phrase are rejected. All accepted articles at this stage (both full and partial review) are made available on the CEA Registry website with the following variables: country, disease, intervention description, intervention type, and comparator description.

Two readers with training in decision analysis and cost-effectiveness analysis independently review each article accepted for full review and record information using a standardized set of forms and instructions. The two readers convene for a consensus audit to resolve any potential discrepancies. On occasion, a third reader may be called upon to help settle disputed items. Finally, data are quality checked by the CEA Registry team and a series of data cleaning steps are performed. Full data extraction is uploaded to the CEA Registry three times a year.

Table A1: Quality of cost-per-QALY and cost-per-DALY studies

Quality indicator	QALY (n=125)	DALY (n=45)
Clearly stated perspective, % yes	83.8	75.6
Discounted costs and QALYs (DALYs), % yes	79.0	64.4
Stated time horizon, % yes	89.5	77.8
Stated year of currency, % yes	82.7	82.2
Conducted incremental analysis correctly, % yes	80.0	57.8
Quality score, mean (SD)	5.04 (1.17)	4.70 (0.97)

QALY = quality-adjusted life year; DALY = disability-adjusted life year; SD = standard deviation

Figure A1: Cost-effectiveness plane showing ICERs from cost-per-QALY studies with a primary geography of China

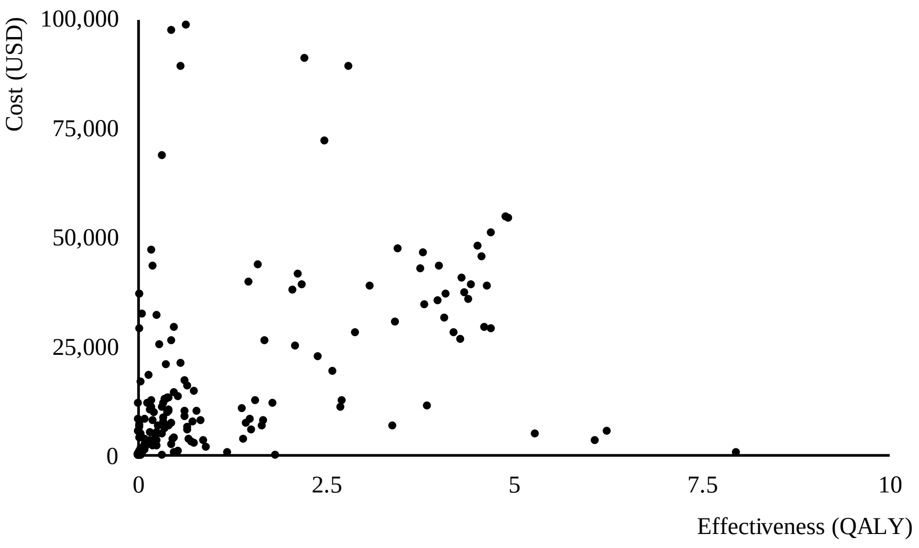

ICER = incremental cost-effectiveness ratio, QALY = quality-adjusted life year; Note: Only northeast quadrant (interventions that were more costly and more effective than the comparator) are shown. ICERs with incremental QALYs of >10 or incremental costs of >USD100,000 are not displayed.

Included studies

1. Zhuang GH, Pan XJ, Wang XL. A cost-effectiveness analysis of universal childhood hepatitis A vaccination in China. Vaccine. 2008;26(35):4608-16.
2. Xie X, Vondeling H. Cost-utility analysis of intensive blood glucose control with metformin versus usual care in overweight type 2 diabetes mellitus patients in Beijing, P.R. China. Value in health : the journal of the International Society for Pharmacoeconomics and Outcomes Research. 2008;11 Suppl 1:S23-32.
3. Yuan Y, Iloeje U, Li H, Hay J, Yao GB. Economic implications of entecavir treatment in suppressing viral replication in chronic hepatitis B (CHB) patients in China from a perspective of the Chinese Social Security program. Value in health : the journal of the International Society for Pharmacoeconomics and Outcomes Research. 2008;11 Suppl 1:S11-22.
4. Palmer JL, Gibbs M, Scheijbeler HW, Kotchie RW, Nielsen S, White J, et al. Cost-effectiveness of switching to biphasic insulin aspart in poorly-controlled type 2 diabetes patients in China. Advances in therapy. 2008;25(8):752-74.
5. Lansingh VC, Carter MJ. Use of Global Visual Acuity Data in a time trade-off approach to calculate the cost utility of cataract surgery. Archives of ophthalmology (Chicago, Ill : 1960). 2009;127(9):1183-93.
6. Hutton DW, So SK, Brandeau ML. Cost-effectiveness of nationwide hepatitis B catch-up vaccination among children and adolescents in China. Hepatology (Baltimore, Md). 2010;51(2):405-14.
7. Liubao P, Xiaomin W, Chongqing T, Karnon J, Gannong C, Jianhe L, et al. Cost-effectiveness analysis of adjuvant therapy for operable breast cancer from a Chinese perspective: doxorubicin plus cyclophosphamide versus docetaxel plus cyclophosphamide. PharmacoEconomics. 2009;27(10):873-86.
8. Chen W, Jiang Z, Shao Z, Sun Q, Shen K. An economic evaluation of adjuvant trastuzumab therapy in HER2-positive early breast cancer. Value in health : the journal of the International Society for Pharmacoeconomics and Outcomes Research. 2009;12 Suppl 3:S82-4.
9. Chen Z, Wang C, Xu X, Feng W. Cost-effectiveness study comparing imatinib with interferon-alpha for patients with newly diagnosed chronic-phase (CP) chronic myeloid leukemia (CML) from the Chinese public health-care system perspective (CPHSP). Value in health : the journal of the International Society for Pharmacoeconomics and Outcomes Research. 2009;12 Suppl 3:S85-8.
10. Wu B, Li T, Chen H, Shen J. Cost-effectiveness of nucleoside analog therapy for hepatitis B in China: a Markov analysis. Value in health : the journal of the International Society for Pharmacoeconomics and Outcomes Research. 2010;13(5):592-600.
11. Palmer JL, Beaudet A, White J, Plun-Favreau J, Smith-Palmer J. Cost-effectiveness of biphasic insulin aspart versus insulin glargine in patients with type 2 diabetes in China. Advances in therapy. 2010;27(11):814-27.
12. Wu B, Chen H, Shen J, Ye M. Cost-effectiveness of adding rh-endostatin to first-line chemotherapy in patients with advanced non-small-cell lung cancer in China. Clinical therapeutics. 2011;33(10):1446-55.
13. Han X, Xu J, Chu Z, Dai D, Lu C, Wang X, et al. Screening acute HIV infections among Chinese men who have sex with men from voluntary counseling & testing centers. PloS one. 2011;6(12):e28792.
14. Zhou L, Guan P, Sun LP, He QC, Yuan Y, Zhou BS. Health economic assessment for screening of gastric cancer in a high risk population in northeastern china. Chinese journal of cancer research = Chung-kuo yen cheng yen chiu. 2011;23(1):21-4.
15. Yang L, Christensen T, Sun F, Chang J. Cost-effectiveness of switching patients with type 2 diabetes from insulin glargine to insulin detemir in Chinese setting: a health economic model based on the PREDICTIVE study. Value in health : the journal of the International Society for Pharmacoeconomics and Outcomes Research. 2012;15(1 Suppl):S56-9.
16. Liu J, Wang Y, Zhang D, Liu B, Ou Q. Comparison of survival and quality of life of hepatectomy and thrombectomy using total hepatic vascular exclusion and chemotherapy alone in

patients with hepatocellular carcinoma and tumor thrombi in the inferior vena cava and hepatic vein. European journal of gastroenterology & hepatology. 2012;24(2):186-94.
17. Wu B, Ye M, Chen H, Shen JF. Costs of trastuzumab in combination with chemotherapy for HER2-positive advanced gastric or gastroesophageal junction cancer: an economic evaluation in the Chinese context. Clinical therapeutics. 2012;34(2):468-79.
18. Wu B, Dong B, Xu Y, Zhang Q, Shen J, Chen H, et al. Economic evaluation of first-line treatments for metastatic renal cell carcinoma: a cost-effectiveness analysis in a health resource-limited setting. PloS one. 2012;7(3):e32530.
19. Wu B, Miao Y, Bai Y, Ye M, Xu Y, Chen H, et al. Subgroup economic analysis for glioblastoma in a health resource-limited setting. PloS one. 2012;7(4):e34588.
20. Gao L, Zhao FL, Li SC. Cost-utility analysis of liraglutide versus glimepiride as add-on to metformin in type 2 diabetes patients in China. International journal of technology assessment in health care. 2012;28(4):436-44.
21. Bai Y, Ye M, Cao H, Ma X, Xu Y, Wu B. Economic evaluation of radiotherapy for early breast cancer after breast-conserving surgery in a health resource-limited setting. Breast cancer research and treatment. 2012;136(2):547-57.
22. Li J, Gilmour S, Zhang H, Koyanagi A, Shibuya K. The epidemiological impact and cost-effectiveness of HIV testing, antiretroviral treatment and harm reduction programs. AIDS (London, England). 2012;26(16):2069-78.
23. Wu B, Wilson A, Wang FF, Wang SL, Wallace DJ, Weisman MH, et al. Cost effectiveness of different treatment strategies in the treatment of patients with moderate to severe rheumatoid arthritis in china. PloS one. 2012;7(10):e47373.
24. Wu B, Shen J, Cheng H. Cost-effectiveness analysis of different rescue therapies in patients with lamivudine-resistant chronic hepatitis B in China. BMC health services research. 2012;12:385.
25. Zeng X, Peng L, Li J, Chen G, Tan C, Wang S, et al. Cost-effectiveness of continuation maintenance pemetrexed after cisplatin and pemetrexed chemotherapy for advanced nonsquamous non-small-cell lung cancer: estimates from the perspective of the Chinese health care system. Clinical therapeutics. 2013;35(1):54-65.
26. Zhu J, Li T, Wang X, Ye M, Cai J, Xu Y, et al. Gene-guided gefitinib switch maintenance therapy for patients with advanced EGFR mutation-positive non-small cell lung cancer: an economic analysis. BMC cancer. 2013;13:39.
27. Lu SQ, McGhee SM, Xie X, Cheng J, Fielding R. Economic evaluation of universal newborn hepatitis B vaccination in China. Vaccine. 2013;31(14):1864-9.
28. Wang S, Peng L, Li J, Zeng X, Ouyang L, Tan C, et al. A trial-based cost-effectiveness analysis of erlotinib alone versus platinum-based doublet chemotherapy as first-line therapy for Eastern Asian nonsquamous non-small-cell lung cancer. PloS one. 2013;8(3):e55917.
29. Chongqing T, Liubao P, Xiaohui Z, Jianhe L, Xiaomin W, Gannong C, et al. Cost-utility analysis of the newly recommended adjuvant chemotherapy for resectable gastric cancer patients in the 2011 Chinese National Comprehensive Cancer Network (NCCN) Clinical Practice Guidelines in Oncology: Gastric Cancer. PharmacoEconomics. 2014;32(3):235-43.
30. Liu X, Li C, Gong H, Cui Z, Fan L, Yu W, et al. An economic evaluation for prevention of diabetes mellitus in a developing country: a modelling study. BMC Public Health. 2013;13:729.
31. Tan C, Peng L, Zeng X, Li J, Wan X, Chen G, et al. Economic evaluation of first-line adjuvant chemotherapies for resectable gastric cancer patients in China. PloS one. 2013;8(12):e83396.
32. Wang M, Moran AE, Liu J, Coxson PG, Heidenreich PA, Gu D, et al. Cost-effectiveness of optimal use of acute myocardial infarction treatments and impact on coronary heart disease mortality in China. Circulation Cardiovascular quality and outcomes. 2014;7(1):78-85.
33. Zeng X, Li J, Peng L, Wang Y, Tan C, Chen G, et al. Economic outcomes of maintenance gefitinib for locally advanced/metastatic non-small-cell lung cancer with unknown EGFR mutations: a semi-Markov model analysis. PloS one. 2014;9(2):e88881.
34. Li D, Yuan Y, Sun LP, Fang X, Zhou BS. Health economics evaluation of a gastric cancer early detection and treatment program in China. Asian Pacific journal of cancer prevention : APJCP. 2014;15(13):5133-6.

35. Wen F, Yao K, Du ZD, He XF, Zhang PF, Tang RL, et al. Cost-effectiveness analysis of colon cancer treatments from MOSIAC and No. 16968 trials. World journal of gastroenterology. 2014;20(47):17976-84.
36. Wu B, Li T, Cai J, Xu Y, Zhao G. Cost-effectiveness analysis of adjuvant chemotherapies in patients presenting with gastric cancer after D2 gastrectomy. BMC cancer. 2014;14:984.
37. Jia Y, Li L, Cui F, Zhang D, Zhang G, Wang F, et al. Cost-effectiveness analysis of a hepatitis B vaccination catch-up program among children in Shandong Province, China. Human vaccines & immunotherapeutics. 2014;10(10):2983-91.
38. Wu B, Song Y, Leng L, Bucala R, Lu LJ. Treatment of moderate rheumatoid arthritis with different strategies in a health resource-limited setting: a cost-effectiveness analysis in the era of biosimilars. Clinical and experimental rheumatology. 2015;33(1):20-6.
39. Pan Y, Chen Q, Zhao X, Liao X, Wang C, Du W, et al. Cost-effectiveness of thrombolysis within 4.5 hours of acute ischemic stroke in China. PloS one. 2014;9(10):e110525.
40. Ma Y, Ying X, Zou H, Xu X, Liu H, Bai L, et al. Rhegmatogenous retinal detachment surgery in elderly people over 70 years old: visual acuity, quality of life, and cost-utility values. PloS one. 2014;9(10):e110256.
41. Liu JY, You RX, Guo M, Zeng L, Zhou P, Zhu L, et al. Tacrolimus Versus Cyclosporine as Primary Immunosuppressant After Renal Transplantation: A Meta-Analysis and Economics Evaluation. American journal of therapeutics. 2016;23(3):e810-24.
42. Zhang Z, Zhai J, Wei Q, Qi J, Guo X, Zhao J. Cost-effectiveness analysis of psychosocial intervention for early stage schizophrenia in China: a randomized, one-year study. BMC psychiatry. 2014;14:212.
43. Toy M, Salomon JA, Jiang H, Gui H, Wang H, Wang J, et al. Population health impact and cost-effectiveness of monitoring inactive chronic hepatitis B and treating eligible patients in Shanghai, China. Hepatology (Baltimore, Md). 2014;60(1):46-55.
44. Feng WM, Bao Y, Tang CW, Fei MY, Guo HH, Tao YL, et al. Optimal selection of methods for mini-invasive treatment of extrahepatic bile duct stones. Hepato-gastroenterology. 2014;61(130):299-303.
45. Pan Y, Wang A, Liu G, Zhao X, Meng X, Zhao K, et al. Cost-effectiveness of clopidogrel-aspirin versus aspirin alone for acute transient ischemic attack and minor stroke. Journal of the American Heart Association. 2014;3(3):e000912.
46. Che D, Zhou H, He J, Wu B. Modeling the impact of the 7-valent pneumococcal conjugate vaccine in Chinese infants: an economic analysis of a compulsory vaccination. BMC health services research. 2014;14:56.
47. Wu B, Kun L, Liu X, He B. Cost-effectiveness of different strategies for stroke prevention in patients with atrial fibrillation in a health resource-limited setting. Cardiovascular drugs and therapy. 2014;28(1):87-98.
48. Ma Y, Ying X, Zou H, Xu X, Liu H, Bai L, et al. Cost-utility analysis of rhegmatogenous retinal detachment surgery in Shanghai, China. Ophthalmic epidemiology. 2015;22(1):13-9.
49. Xu X, Ma YY, Zou HD. Cost-Utility Analysis of Cataract Surgery in Advanced Glaucoma Patients. Journal of glaucoma. 2016;25(7):e657-62.
50. Gu D, He J, Coxson PG, Rasmussen PW, Huang C, Thanataveerat A, et al. The Cost-Effectiveness of Low-Cost Essential Antihypertensive Medicines for Hypertension Control in China: A Modelling Study. PLoS medicine. 2015;12(8):e1001860.
51. Caldwell R, Roberts CS, An Z, Chen CI, Wang B. The health and economic impact of vaccination with 7-valent pneumococcal vaccine (PCV7) during an annual influenza epidemic and influenza pandemic in China. BMC infectious diseases. 2015;15:284.
52. Deng J, Gu S, Shao H, Dong H, Zou D, Shi L. Cost-effectiveness analysis of exenatide twice daily (BID) vs insulin glargine once daily (QD) as add-on therapy in Chinese patients with Type 2 diabetes mellitus inadequately controlled by oral therapies. Journal of medical economics. 2015;18(11):974-89.
53. Yin J, Ji Z, Liang P, Wu Q, Cui F, Wang F, et al. The doses of 10 mug should replace the doses of 5 mug in newborn hepatitis B vaccination in China: A cost-effectiveness analysis. Vaccine. 2015;33(31):3731-8.

54. Dranitsaris G, Yu B, King J, Kaura S, Zhang A. Nab-paclitaxel, docetaxel, or solvent-based paclitaxel in metastatic breast cancer: a cost-utility analysis from a Chinese health care perspective. ClinicoEconomics and outcomes research : CEOR. 2015;7:249-56.
55. Hur C, Choi SE, Kong CY, Wang GQ, Xu H, Polydorides AD, et al. High-resolution microendoscopy for esophageal cancer screening in China: A cost-effectiveness analysis. World journal of gastroenterology. 2015;21(18):5513-23.
56. Li T, Liu M, Ben H, Xu Z, Zhong H, Wu B. Clopidogrel versus aspirin in patients with recent ischemic stroke and established peripheral artery disease: an economic evaluation in a Chinese setting. Clinical drug investigation. 2015;35(6):365-74.
57. Gu S, Deng J, Shi L, Mu Y, Dong H. Cost-effectiveness of saxagliptin vs glimepiride as a second-line therapy added to metformin in Type 2 diabetes in China. Journal of medical economics. 2015;18(10):808-20.
58. Zhang P, Yang Y, Wen F, He X, Tang R, Du Z, et al. Cost-effectiveness of sorafenib as a first-line treatment for advanced hepatocellular carcinoma. European journal of gastroenterology & hepatology. 2015;27(7):853-9.
59. Zhou J, Zhao R, Wen F, Zhang P, Tang R, Du Z, et al. Cost-effectiveness analysis of gemcitabine, S-1 and gemcitabine plus S-1 for treatment of advanced pancreatic cancer based on GEST study. Medical oncology (Northwood, London, England). 2015;32(4):121.
60. Zhang C, Ke W, Gao Y, Zhou S, Liu L, Ye X, et al. Cost-effectiveness analysis of antiviral therapies for hepatitis B e antigen-positive chronic hepatitis B patients in China. Clinical drug investigation. 2015;35(3):197-209.
61. Jiang Y, Ni W. Expected lifetime numbers, risks, and burden of osteoporotic fractures for 50-year old Chinese women: a discrete event simulation incorporating FRAX. Journal of bone and mineral metabolism. 2016;34(6):714-22.
62. Wu B, Li J, Wu H. Strategies to Screen for Diabetic Retinopathy in Chinese Patients with Newly Diagnosed Type 2 Diabetes: A Cost-Effectiveness Analysis. Medicine. 2015;94(45):e1989.
63. Toy M, Hutton DW, So SK. Cost-Effectiveness and Cost Thresholds of Generic and Brand Drugs in a National Chronic Hepatitis B Treatment Program in China. PloS one. 2015;10(11):e0139876.
64. Wan X, Peng L, Ma J, Chen G, Li Y. Subgroup Economic Evaluation of Radiotherapy for Breast Cancer After Mastectomy. Clinical therapeutics. 2015;37(11):2515-26.e5.
65. Wen F, Yang Y, Zhang P, Zhang J, Zhou J, Tang R, et al. Cost-effectiveness of RAS screening before monoclonal antibodies therapy in metastatic colorectal cancer based on FIRE3 Study. Cancer biology & therapy. 2015;16(11):1577-84.
66. Duan L, Huang M, Yan H, Zhang Y, Gu F. Cost-effectiveness analysis of two therapeutic schemes in the treatment of acromegaly: a retrospective study of 168 cases. Journal of endocrinological investigation. 2015;38(7):717-23.
67. Xuan JW, Song RL, Xu GX, Lu WQ, Lu YJ, Liu Z. Modeling the cost-effectiveness of ilaprazole versus omeprazole for the treatment of newly diagnosed duodenal ulcer patients in China. Journal of medical economics. 2016;19(11):1056-60.
68. Wu B, Li J, Lin H, Wu H. Different Strategies for the Treatment of Age-Related Macular Degeneration in China: An Economic Evaluation. Journal of ophthalmology. 2016;2016:7689862.
69. Tang Q, Sun Z, Zhang N, Xu G, Song P, Xu L, et al. Cost-Effectiveness of Bariatric Surgery for Type 2 Diabetes Mellitus: A Randomized Controlled Trial in China. Medicine. 2016;95(20):e3522.
70. Goyal A, Murray JM. Cost-Effectiveness of Peg-Interferon, Interferon and Oral Nucleoside Analogues in the Treatment of Chronic Hepatitis B and D Infections in China. Clinical drug investigation. 2016;36(8):637-48.
71. Zheng HR, Wen F, Wu YF, Wheeler JRC, Li Q. Cost-effectiveness analysis of additional docetaxel for metastatic hormone-sensitive prostate cancer treated with androgen-deprivation therapy from a Chinese perspective. European journal of cancer care. 2017;26(6).
72. Zhang P, Yang Y, Wen F, Wheeler J, Fu P, Li Q. Cost-effectiveness analysis of antiviral therapy in patients with advanced hepatitis B virus-related hepatocellular carcinoma treated with sorafenib. Journal of gastroenterology and hepatology. 2016;31(12):1978-85.

73. Chen M, Si L, Winzenberg TM, Gu J, Jiang Q, Palmer AJ. Cost-effectiveness of raloxifene in the treatment of osteoporosis in Chinese postmenopausal women: impact of medication persistence and adherence. Patient preference and adherence. 2016;10:415-23.
74. Shen K, Xiong T, Tan SC, Wu J. Oseltamivir Treatment for Children with Influenza-Like Illness in China: A Cost-Effectiveness Analysis. PloS one. 2016;11(4):e0153664.
75. Zhou J, Zhao R, Wen F, Zhang P, Wu Y, Tang R, et al. Cost-effectiveness analysis of treatments for metastatic pancreatic cancer based on PRODIGE and MPACT trials. Tumori. 2016;2016(3):294-300.
76. Wang W, Wang J, Dang S, Zhuang G. Cost-effectiveness of antiviral therapy during late pregnancy to prevent perinatal transmission of hepatitis B virus. PeerJ. 2016;4:e1709.
77. Zhang C, Ke W, Liu L, Gao Y, Yao Z, Ye X, et al. Cost-effectiveness comparison of lamivudine plus adefovir combination treatment and nucleos(t)ide analog monotherapies in Chinese chronic hepatitis B patients. Drug design, development and therapy. 2016;10:897-910.
78. Zhang C, Zhang H, Shi J, Wang D, Zhang X, Yang J, et al. Trial-Based Cost-Utility Analysis of Icotinib versus Gefitinib as Second-Line Therapy for Advanced Non-Small Cell Lung Cancer in China. PloS one. 2016;11(3):e0151846.
79. Zhao Y, Zhang X, Zhu F, Jin H, Wang B. A preliminary cost-effectiveness analysis of hepatitis E vaccination among pregnant women in epidemic regions. Human vaccines & immunotherapeutics. 2016;12(8):2003-9.
80. Chai Y, Wang J, Wang T, Shi F, Wang J, Su J, et al. Cost-effectiveness of radical hysterectomy with adjuvant radiotherapy versus radical radiotherapy for FIGO stage IIB cervical cancer. OncoTargets and therapy. 2016;9:349-54.
81. Wang M, Moran AE, Liu J, Coxson PG, Penko J, Goldman L, et al. Projected Impact of Salt Restriction on Prevention of Cardiovascular Disease in China: A Modeling Study. PloS one. 2016;11(2):e0146820.
82. Si L, Winzenberg TM, Chen M, Jiang Q, Neil A, Palmer AJ. Screening for osteoporosis in Chinese post-menopausal women: a health economic modelling study. Osteoporosis international : a journal established as result of cooperation between the European Foundation for Osteoporosis and the National Osteoporosis Foundation of the USA. 2016;27(7):2259-69.
83. Ma Y, Huang J, Zhu B, Sun Q, Miao Y, Zou H. Cost-Utility Analyses of Cataract Surgery in Advanced Age-Related Macular Degeneration. Optometry and vision science : official publication of the American Academy of Optometry. 2016;93(2):165-72.
84. Zhang P, Wen F, Fu P, Yang Y, Li Q. Addition of docetaxel and/or zoledronic acid to standard of care for hormone-naive prostate cancer: a cost-effectiveness analysis. Tumori. 2017;103(4):380-6.
85. Yan X, Gu X, Xu Z, Lin H, Wu B. Cost-Effectiveness of Different Strategies for the Prevention of Venous Thromboembolism After Total Hip Replacement in China. Advances in therapy. 2017;34(2):466-80.
86. Li J, Wen Z, Cai A, Tian F, Zhang L, Luo X, et al. Real-world cost-effectiveness of infliximab for moderate-to-severe rheumatoid arthritis in a medium-sized city of China. Journal of comparative effectiveness research. 2017;6(3):205-18.
87. Sheng G, Chen S, Dong C, Zhang R, Miao M, Wu D, et al. Societal implications of medical insurance coverage for imatinib as first-line treatment of chronic myeloid leukemia in China: a cost-effectiveness analysis. Journal of medical economics. 2017;20(4):371-81.
88. Lai K, Zhang C, Ke W, Gao Y, Zhou S, Liu L, et al. Cost-Effectiveness Comparison Between the Response-Guided Therapies and Monotherapies of Nucleos(t)ide Analogues for Chronic Hepatitis B Patients in China. Clinical drug investigation. 2017;37(3):233-47.
89. Zang X, Tang H, Min JE, Gu D, Montaner JS, Wu Z, et al. Cost-Effectiveness of the 'One4All' HIV Linkage Intervention in Guangxi Zhuang Autonomous Region, China. PloS one. 2016;11(11):e0167308.
90. Yue WW, Wang SR, Li XL, Xu HX, Lu F, Sun LP, et al. Quality of Life and Cost-Effectiveness of Radiofrequency Ablation versus Open Surgery for Benign Thyroid Nodules: a retrospective cohort study. Scientific reports. 2016;6:37838.
91. Gu S, Zeng Y, Yu D, Hu X, Dong H. Cost-Effectiveness of Saxagliptin versus Acarbose as Second-Line Therapy in Type 2 Diabetes in China. PloS one. 2016;11(11):e0167190.

92. Cui S, Tobe RG, Mo X, Liu X, Xu L, Li S. Cost-effectiveness analysis of rotavirus vaccination in China: Projected possibility of scale-up from the current domestic option. BMC infectious diseases. 2016;16(1):677.
93. Zhao D, Gai Tobe R, Cui M, He J, Wu B. Cost-effectiveness of a 23-valent pneumococcal polysaccharide vaccine immunization programme for the elderly in Shanghai, China. Vaccine. 2016;34(50):6158-65.
94. Wen F, Zheng H, Wu Y, Wheeler J, Zeng X, Fu P, et al. Cost-effectiveness Analysis of Fluorouracil, Leucovorin, and Irinotecan versus Epirubicin, Cisplatin, and Capecitabine in Patients with Advanced Gastric Adenocarcinoma. Scientific reports. 2016;6:36060.
95. Shao H, Zhai S, Zou D, Mir MU, Zawadzki NK, Shi Q, et al. Cost-effectiveness analysis of dapagliflozin versus glimepiride as monotherapy in a Chinese population with type 2 diabetes mellitus. Current medical research and opinion. 2017;33(2):359-69.
96. Maurer KA, Chen HF, Wagner AL, Hegde ST, Patel T, Boulton ML, et al. Cost-effectiveness analysis of pneumococcal vaccination for infants in China. Vaccine. 2016;34(50):6343-9.
97. Gu S, Mu Y, Zhai S, Zeng Y, Zhen X, Dong H. Cost-Effectiveness of Dapagliflozin versus Acarbose as a Monotherapy in Type 2 Diabetes in China. PloS one. 2016;11(11):e0165629.
98. Chen HD, Zhou J, Wen F, Zhang PF, Zhou KX, Zheng HR, et al. Cost-effectiveness analysis of apatinib treatment for chemotherapy-refractory advanced gastric cancer. Journal of cancer research and clinical oncology. 2017;143(2):361-8.
99. Ke W, Zhang C, Liu L, Gao Y, Yao Z, Ye X, et al. Cost-effectiveness analysis of tenofovir disoproxil fumarate for treatment of chronic hepatitis B in China. Hepatology international. 2016;10(6):924-36.
100. Mo X, Gai Tobe R, Liu X, Mori R. Cost-effectiveness and Health Benefits of Pediatric 23-valent Pneumococcal Polysaccharide Vaccine, 7-valent Pneumococcal Conjugate Vaccine and Forecasting 13-valent Pneumococcal Conjugate Vaccine in China. The Pediatric infectious disease journal. 2016;35(11):e353-e61.
101. Yang L, Chuen Tan S, Chen C, Wang X, Li X, Yang X. Economic Evaluation of Sevelamer versus Calcium-based Binders in Treating Hyperphosphatemia among Patients with End-stage Renal Disease in China. Clinical therapeutics. 2016;38(11):2459-67.e1.
102. Azuara-Blanco A, Burr J, Ramsay C, Cooper D, Foster PJ, Friedman DS, et al. Effectiveness of early lens extraction for the treatment of primary angle-closure glaucoma (EAGLE): a randomised controlled trial. Lancet (London, England). 2016;388(10052):1389-97.
103. Ni W, Jiang Y. Evaluation on the cost-effective threshold of osteoporosis treatment on elderly women in China using discrete event simulation model. Osteoporosis international : a journal established as result of cooperation between the European Foundation for Osteoporosis and the National Osteoporosis Foundation of the USA. 2017;28(2):529-38.
104. Yan X, Gu X, Zhou L, Lin H, Wu B. Cost Effectiveness of Apixaban and Enoxaparin for the Prevention of Venous Thromboembolism After Total Knee Replacement in China. Clinical drug investigation. 2016;36(12):1001-10.
105. Zhang H, Huo M, Chao J, Liu P. Application of Bayesian Approach to Cost-Effectiveness Analysis of Antiviral Treatments in Chronic Hepatitis B. PloS one. 2016;11(8):e0161936.
106. Zhang P, Wen F, Li Q. FOLFOX4 or sorafenib as the first-line treatments for advanced hepatocellular carcinoma: A cost-effectiveness analysis. Digestive and liver disease : official journal of the Italian Society of Gastroenterology and the Italian Association for the Study of the Liver. 2016;48(12):1492-7.
107. Zhou J, Zhao R, Wen F, Zhang P, Tang R, Chen H, et al. Economic evaluation study (CHEER-compliant): Cost-effectiveness analysis of RAS screening for treatment of metastatic colorectal cancer based on the CALGB 80405 trial. Medicine. 2016;95(27):e3762.
108. Cui M, Tu CC, Chen EZ, Wang XL, Tan SC, Chen C. A Cost-Effectiveness Analysis of Clopidogrel for Patients with Non-ST-Segment Elevation Acute Coronary Syndrome in China. Advances in therapy. 2016;33(9):1600-11.
109. Zhang X, Liu S, Li Y, Wang Y, Tian M, Liu G. Long-Term Effectiveness and Cost-Effectiveness of Metformin Combined with Liraglutide or Exenatide for Type 2 Diabetes Mellitus Based on the CORE Diabetes Model Study. PloS one. 2016;11(6):e0156393.

110. Chen GF, Wei L, Chen J, Duan ZP, Dou XG, Xie Q, et al. Will Sofosbuvir/Ledipasvir (Harvoni) Be Cost-Effective and Affordable for Chinese Patients Infected with Hepatitis C Virus? An Economic Analysis Using Real-World Data. PloS one. 2016;11(6):e0155934.
111. Lu S, Zhang J, Ye M, Wang B, Wu B. Economic analysis of ALK testing and crizotinib therapy for advanced non-small-cell lung cancer. Pharmacogenomics. 2016;17(9):985-94.
112. Du Q, Zhai Q, Zhu B, Xu XL, Yu B. Economic evaluation of 5-HT3 receptor antagonists in combination with dexamethasone for the prevention of 'overall' nausea and vomiting following highly emetogenic chemotherapy in Chinese adult patients. Journal of oncology pharmacy practice : official publication of the International Society of Oncology Pharmacy Practitioners. 2017;23(6):403-12.
113. Wu JT, Jit M, Zheng Y, Leung K, Xing W, Yang J, et al. Routine Pediatric Enterovirus 71 Vaccination in China: a Cost-Effectiveness Analysis. PLoS medicine. 2016;13(3):e1001975.
114. Chen T, Yu D, Cornelius V, Qin R, Cai Y, Jiang Z, et al. Potential health impact and cost-effectiveness of drug therapy for prehypertension. International journal of cardiology. 2017;240:403-8.
115. Wu B, Yao Y, Zhang K, Ma X. RAS testing and cetuximab treatment for metastatic colorectal cancer: a cost-effectiveness analysis in a setting with limited health resources. Oncotarget. 2017;8(41):71164-72.
116. Gu S, Wang X, Qiao Q, Gao W, Wang J, Dong H. Cost-effectiveness of exenatide twice daily vs insulin glargine as add-on therapy to oral antidiabetic agents in patients with type 2 diabetes in China. Diabetes, obesity & metabolism. 2017;19(12):1688-97.
117. Cheng X, Zhao Y, Zhang X, Jin H, Min J. Health economic evaluation of immunization strategies of hepatitis E vaccine for elderly population. Human vaccines & immunotherapeutics. 2017;13(8):1873-8.
118. Wang H, Yang L, Wang F, Zhang L. Strategies and cost-effectiveness evaluation of persistent albuminuria screening among high-risk population of chronic kidney disease. BMC nephrology. 2017;18(1):135.
119. Chen H, Chen L. Estimating cost-effectiveness associated with all-oral regimen for chronic hepatitis C in China. PloS one. 2017;12(4):e0175189.
120. Qiu J, Yu C, Ariyaratne TV, Foteff C, Ke Z, Sun Y, et al. Cost-Effectiveness of Pediatric Cochlear Implantation in Rural China. Otology & neurotology : official publication of the American Otological Society, American Neurotology Society [and] European Academy of Otology and Neurotology. 2017;38(6):e75-e84.
121. Zhou K, Wen F, Zhang P, Zhou J, Chen H, Zheng H, et al. Efficacy and cost-effectiveness of second-line chemotherapy in elderly patients with advanced gastric cancer. Clinical & translational oncology : official publication of the Federation of Spanish Oncology Societies and of the National Cancer Institute of Mexico. 2017;19(9):1117-24.
122. Zhu B, Ma Y, Zou H. Cost-utility analyses of cataract surgery in vision-threatening diabetic retinopathy. Journal of cataract and refractive surgery. 2017;43(1):95-101.
123. Wan XM, Peng LB, Ma JA, Li YJ. Economic evaluation of nivolumab as a second-line treatment for advanced renal cell carcinoma from US and Chinese perspectives. Cancer. 2017;123(14):2634-41.
124. Wu B, Zhang S, Lin H, Mou S. Prevention of renal failure in Chinese patients with newly diagnosed type 2 diabetes: A cost-effectiveness analysis. Journal of diabetes investigation. 2018;9(1):152-61.
125. Lin S, Ye Q, Wang M, Wu Y, Weng Z, Zhu Y. Antiviral Therapy in Chronic Hepatitis B With Mild Acute Exacerbation. Gastroenterology research. 2017;10(1):6-14.
126. Miller MA. An assessment of the value of Haemophilus influenzae type b conjugate vaccine in Asia. The Pediatric infectious disease journal. 1998;17(9 Suppl):S152-9.
127. Xu Q, Jin SG, Zhang LX. Cost effectiveness of DOTS and non-DOTS strategies for smear-positive pulmonary tuberculosis in Beijing. Biomedical and environmental sciences : BES. 2000;13(4):307-13.
128. Xu Q, Wu ZL, Jin SG, Zhang LX. Tuberculosis control priorities defined by using cost-effectiveness and burden of disease. Biomedical and environmental sciences : BES. 2002;15(2):172-6.

129. Ding D, Kilgore PE, Clemens JD, Wei L, Zhi-Yi X. Cost-effectiveness of routine immunization to control Japanese encephalitis in Shanghai, China. Bulletin of the World Health Organization. 2003;81(5):334-42.
130. Dziekan G, Chisholm D, Johns B, Rovira J, Hutin YJ. The cost-effectiveness of policies for the safe and appropriate use of injection in healthcare settings. Bulletin of the World Health Organization. 2003;81(4):277-85.
131. Budke CM, Jiamin Q, Qian W, Torgerson PR. Economic effects of echinococcosis in a disease-endemic region of the Tibetan Plateau. The American journal of tropical medicine and hygiene. 2005;73(1):2-10.
132. Chisholm D. Cost-effectiveness of first-line antiepileptic drug treatments in the developing world: a population-level analysis. Epilepsia. 2005;46(5):751-9.
133. Woo PP, Kim JJ, Leung GM. What is the most cost-effective population-based cancer screening program for Chinese women? Journal of clinical oncology : official journal of the American Society of Clinical Oncology. 2007;25(6):617-24.
134. Ma G, Jin Y, Li Y, Zhai F, Kok FJ, Jacobsen E, et al. Iron and zinc deficiencies in China: what is a feasible and cost-effective strategy? Public health nutrition. 2008;11(6):632-8.
135. Goldie SJ, Diaz M, Kim SY, Levin CE, Van Minh H, Kim JJ. Mathematical models of cervical cancer prevention in the Asia Pacific region. Vaccine. 2008;26 Suppl 12:M17-29.
136. Stevenson M, Yu J, Hendrie D, Li LP, Ivers R, Zhou Y, et al. Reducing the burden of road traffic injury: translating high-income country interventions to middle-income and low-income countries. Injury prevention : journal of the International Society for Child and Adolescent Injury Prevention. 2008;14(5):284-9.
137. Fiedler JL, Macdonald B. A strategic approach to the unfinished fortification agenda: feasibility, costs, and cost-effectiveness analysis of fortification programs in 48 countries. Food and nutrition bulletin. 2009;30(4):283-316.
138. Rheingans RD, Antil L, Dreibelbis R, Podewils LJ, Bresee JS, Parashar UD. Economic costs of rotavirus gastroenteritis and cost-effectiveness of vaccination in developing countries. The Journal of infectious diseases. 2009;200 Suppl 1:S16-27.
139. Duintjer Tebbens RJ, Pallansch MA, Cochi SL, Wassilak SG, Linkins J, Sutter RW, et al. Economic analysis of the global polio eradication initiative. Vaccine. 2010;29(2):334-43.
140. Hong FC, Liu JB, Feng TJ, Liu XL, Pan P, Zhou H, et al. Congenital syphilis: an economic evaluation of a prevention program in China. Sexually transmitted diseases. 2010;37(1):26-31.
141. Nakamura MM, Tasslimi A, Lieu TA, Levine O, Knoll MD, Russell LB, et al. Cost effectiveness of child pneumococcal conjugate vaccination in middle-income countries. International health. 2011;3(4):270-81.
142. Wang S, Moss JR, Hiller JE. The cost-effectiveness of HIV voluntary counseling and testing in China. Asia-Pacific journal of public health. 2011;23(4):620-33.
143. De Steur H, Blancquaert D, Gellynck X, Lambert W, Van Der Straeten D. Ex-ante evaluation of biotechnology innovations: the case of folate biofortified rice in China. Current pharmaceutical biotechnology. 2012;13(15):2751-60.
144. Guo Y, Zhang W, Zhang Y, Lin X, Zhang B, Chen C, et al. Cost-effectiveness analysis of preventing mother-to-child transmission of hepatitis B by injecting hepatitis B immune globulin. European journal of gastroenterology & hepatology. 2012;24(12):1363-9.
145. Liu N, Yen C, Fang ZY, Tate JE, Jiang B, Parashar UD, et al. Projected health impact and cost-effectiveness of rotavirus vaccination among children <5 years of age in China. Vaccine. 2012;30(48):6940-5.
146. Yin Z, Beeler Asay GR, Zhang L, Li Y, Zuo S, Hutin YJ, et al. An economic evaluation of the use of Japanese encephalitis vaccine in the expanded program of immunization of Guizhou province, China. Vaccine. 2012;30(37):5569-77.
147. Huang LH, Zhang L, Tobe RY, Qi FH, Sun L, Teng Y, et al. Cost-effectiveness analysis of neonatal hearing screening program in China: should universal screening be prioritized? BMC health services research. 2012;12:97.
148. De Steur H, Gellynck X, Blancquaert D, Lambert W, Van Der Straeten D, Qaim M. Potential impact and cost-effectiveness of multi-biofortified rice in China. New biotechnology. 2012;29(3):432-42.

149. Griffiths UK, Clark A, Hajjeh R. Cost-effectiveness of Haemophilus influenzae type b conjugate vaccine in low- and middle-income countries: regional analysis and assessment of major determinants. The Journal of pediatrics. 2013;163(1 Suppl):S50-S9.e9.
150. Tobe RG, Mori R, Huang L, Xu L, Han D, Shibuya K. Cost-effectiveness analysis of a national neonatal hearing screening program in China: conditions for the scale-up. PloS one. 2013;8(1):e51990.
151. Owusu-Edusei K, Jr., Tao G, Gift TL, Wang A, Wang L, Tun Y, et al. Cost-effectiveness of integrated routine offering of prenatal HIV and syphilis screening in China. Sexually transmitted diseases. 2014;41(2):103-10.
152. Azman AS, Golub JE, Dowdy DW. How much is tuberculosis screening worth? Estimating the value of active case finding for tuberculosis in South Africa, China, and India. BMC medicine. 2014;12:216.
153. Knight GM, Griffiths UK, Sumner T, Laurence YV, Gheorghe A, Vassall A, et al. Impact and cost-effectiveness of new tuberculosis vaccines in low- and middle-income countries. Proceedings of the National Academy of Sciences of the United States of America. 2014;111(43):15520-5.
154. Wang WB, Zhang H, Petzold M, Zhao Q, Xu B, Zhao GM. Cost-effectiveness of the Health X Project for tuberculosis control in China. The international journal of tuberculosis and lung disease : the official journal of the International Union against Tuberculosis and Lung Disease. 2014;18(8):939-45.
155. Marseille E, Jiwani A, Raut A, Verguet S, Walson J, Kahn JG. Scaling up integrated prevention campaigns for global health: costs and cost-effectiveness in 70 countries. BMJ open. 2014;4(6):e003987.
156. Fitzpatrick C, Hui Z, Lixia W, Renzhong L, Yunzhou R, Mingting C, et al. Cost-effectiveness of a comprehensive programme for drug-resistant tuberculosis in China. Bulletin of the World Health Organization. 2015;93(11):775-84.
157. Kuznik A, Muhumuza C, Komakech H, Marques EM, Lamorde M. Antenatal syphilis screening using point-of-care testing in low- and middle-income countries in Asia and Latin America: a cost-effectiveness analysis. PloS one. 2015;10(5):e0127379.
158. Paternina-Caicedo A, De la Hoz-Restrepo F, Alvis-Guzman N. Epidemiological and Economic Impact of Monovalent and Pentavalent Rotavirus Vaccines in Low and Middle Income Countries: A Cost-effectiveness Modeling Analysis. The Pediatric infectious disease journal. 2015;34(7):e176-84.
159. Basu S, Yudkin JS, Sussman JB, Millett C, Hayward RA. Alternative Strategies to Achieve Cardiovascular Mortality Goals in China and India: A Microsimulation of Target- Versus Risk-Based Blood Pressure Treatment. Circulation. 2016;133(9):840-8.
160. Basu S, Shankar V, Yudkin JS. Comparative effectiveness and cost-effectiveness of treat-to-target versus benefit-based tailored treatment of type 2 diabetes in low-income and middle-income countries: a modelling analysis. The lancet Diabetes & endocrinology. 2016;4(11):922-32.
161. Menzies NA, Gomez GB, Bozzani F, Chatterjee S, Foster N, Baena IG, et al. Cost-effectiveness and resource implications of aggressive action on tuberculosis in China, India, and South Africa: a combined analysis of nine models. The Lancet Global health. 2016;4(11):e816-e26.
162. Zhang S, Incardona B, Qazi SA, Stenberg K, Campbell H, Nair H. Cost-effectiveness analysis of revised WHO guidelines for management of childhood pneumonia in 74 Countdown countries. Journal of global health. 2017;7(1):010409.
163. Lu X, Smare C, Kambili C, El Khoury AC, Wolfson LJ. Health outcomes of bedaquiline in the treatment of multidrug-resistant tuberculosis in selected high burden countries. BMC health services research. 2017;17(1):87.
164. Xie Y, Tan X, Shao H, Liu Q, Tou J, Zhang Y, et al. VIA/VILI is more suitable for cervical cancer prevention in Chinese poverty-stricken region: a health economic evaluation. BMC Public Health. 2017;17(1):118.
165. Webb M, Fahimi S, Singh GM, Khatibzadeh S, Micha R, Powles J, et al. Cost effectiveness of a government supported policy strategy to decrease sodium intake: global analysis across 183 nations. Bmj. 2017;356:i6699.

166. Townsend J, Greenland K, Curtis V. Costs of diarrhoea and acute respiratory infection attributable to not handwashing: the cases of India and China. Tropical medicine & international health : TM & IH. 2017;22(1):74-81.
167. Tobe RG, Martin GR, Li F, Moriichi A, Wu B, Mori R. Cost-effectiveness analysis of neonatal screening of critical congenital heart defects in China. Medicine. 2017;96(46):e8683.
168. Wang W, Song J, Wang J, Li Y, Deng H, Li M, et al. Cost-effectiveness of a national enterovirus 71 vaccination program in China. PLoS neglected tropical diseases. 2017;11(9):e0005899.
169. Stover J, Rosen JE, Carvalho MN, Korenromp EL, Friedman HS, Cogan M, et al. The case for investing in the male condom. PloS one. 2017;12(5):e0177108.
170. Zhuang X, Peng P, Sun H, Chu M, Jiang S, Jiang L, et al. Scaling Up Human Immunodeficiency Virus Screening and Antiretroviral Therapy Among Men Who Have Sex With Men to Achieve the 90-90-90 Targets in China. Sexually transmitted diseases. 2018;45(5):343-9.
